# Plasma disappearance rate of albumin when infused as a 20% solution

**DOI:** 10.1186/s13054-022-03979-1

**Published:** 2022-04-11

**Authors:** Markus Zdolsek, Patrick Y. Wuethrich, Michaela Gunnström, Joachim H. Zdolsek, Emma Hasselgren, Christian M. Beilstein, Dominique Engel, Robert G. Hahn

**Affiliations:** 1 Department of Biomedical and Clinical Sciences (BKV), Linköping University, Linköping, Sweden; 2 Department of Anaesthesiology and Pain Medicine, Inselspital, Bern University Hospital, University of Bern, Bern, Switzerland; 3 Perioperative Medicine and Intensive Care, Karolinska University Hospital, Solna, Sweden; 4 Research Unit, Södertälje Hospital, Södertälje, Sweden; 5 Karolinska Institutet at Danderyds Hospital (KIDS), Stockholm, Sweden

**Keywords:** Albumin, Pharmacokinetics, Half-life, C-reactive protein

## Abstract

**Background:**

The transcapillary leakage of albumin is increased by inflammation and major surgery, but whether exogenous albumin also disappears faster is unclear.

**Methods:**

An intravenous infusion of 3 mL/kg of 20% albumin was given over 30 min to 70 subjects consisting of 15 healthy volunteers, 15 post-burn patients, 15 patients who underwent surgery with minor bleeding, 10 who underwent surgery with major bleeding (mean, 1.1 L) and 15 postoperative patients. Blood Hb and plasma albumin were measured on 15 occasions over 5 h. The rate of albumin disappearance from the plasma was quantitated with population kinetic methodology and reported as the half-life (T_1/2_).

**Results:**

No differences were observed for T_1/2_ between volunteers, post-burn patients, patients who underwent surgery with minor bleeding and postoperative patients. The T_1/2_ averaged 16.2 h, which corresponds to 3.8% of the amount infused per h. Two groups showed plasma concentrations of C-reactive protein of approximately 60 mg/L and still had a similarly long T_1/2_ for albumin. By contrast, patients undergoing surgery associated with major hemorrhage had a shorter T_1/2_, corresponding to 15% of the infused albumin per h. In addition, our analyses show that the T_1/2_ differ greatly depending on whether the calculations consider plasma volume changes and blood losses.

**Conclusion:**

The disappearance rate of the albumin in 20% preparations was low in volunteers, in patients with moderately severe inflammation, and in postoperative patients.

**Supplementary Information:**

The online version contains supplementary material available at 10.1186/s13054-022-03979-1.

## Introduction

Circulating albumin leaks from the plasma at a rate of 5% of the plasma pool per h [1, 2], which corresponds to an intravascular half-life (T_1/2_) of 7–10 h. This leakage occurs faster during surgery and in acute inflammation and is believed to be the cause of hypoalbuminemia in severe disease [2].

These issues raise concerns about the clinical efficacy of 20% albumin, which is used for plasma volume expansion in major surgery and intensive care [3, 4] where the endothelial glycocalyx layer is frequently injured [5]. The volume expansion is strongly associated with the increased amount of albumin in the circulation [6, 7] and, therefore, ‘leaky capillaries’ may impair the intravascular persistence of administered albumin [8]. However, this possibility has not been studied with a consistent methodology.

We aimed to determine whether the T_1/2_ of a load of 20% albumin in healthy volunteers differs from the T_1/2_ obtained in four clinical scenarios. Data were pooled from studies performed in different settings, but where the same research protocol had been used [9–12]. Different approaches to calculate T_1/2_ were applied to search for a kinetic model that seemed most appropriate. No radioactive tracer was used.

The hypothesis was that one or several of the studied clinical situations would be associated with a more rapid disappearance of exogenous albumin from the vascular space than was observed in healthy volunteers.

## Methods

### Subjects

The present report is a secondary publication to four publications in which 3 mL/kg of 20% albumin (approximately 200 mL) was administered over 30 min at a constant rate by an infusion pump to 70 subjects in the following clinical settings: healthy volunteers (*N* = 15) [9], post-burn patients (*N* = 15) [10], ongoing surgery with minor blood loss (*N* = 15) [11], ongoing surgery with major bleeding (*N* = 10) [12], and postoperative patients (*N* = 15) [9]. Reporting adhered to the STROBE checklist.

### Inclusion and exclusion criteria

Healthy volunteers included subjects with age > 18 years and blood hemoglobin (Hb) concentration > 100 g/L [9].

The post-burn study recruited patients with a burn area > 6% of the Total Body Surface Area and age between 18 and 80 years. Exclusion criteria were unconsciousness, severe allergy, kidney failure or heart failure [10].

The study of ongoing elective surgery with minor blood loss recruited patients scheduled for surgery lasting for at least 5 h but without an expected major hemorrhage. Exclusion criteria were age < 18 years, poor health status (American Society of Anesthesiologist´s [ASA] Classes III-IV), severe allergy, kidney failure or heart failure [11].

For the study of ongoing elective surgery with major bleeding, patients were recruited who were> 18 years old and scheduled to undergo pelvic lymph node dissection, open radical cystectomy and urinary diversion. Exclusion criteria were renal dysfunction (Kidney Disease Outcomes Quality Initiative stage ≥ 3), severe allergy, heart failure and alcohol abuse [12].

Inclusion criteria for the study of postoperative patients with inflammation were age > 18 years, ASA I-II, and Hb concentration of > 90 g/L [9].

### Health conditions and treatments

The healthy volunteers fasted from midnight but ingested one sandwich and to drink one glass (200 mL) of clear liquid, 2 h before any blood sampling was started between 7.00 and 9.00 AM [9].

The post-burn patients were studied in the morning approximately one week after their burn injury. They were fully conscious, hemodynamically stable and had fasted overnight, but were allowed to ingest one sandwich and drink one glass (200 mL) of liquid 1.5 h prior to the experiment. Some patients were treated with intravenous antibiotics and could have received 100 mL of 0.9% saline as a vehicle during the fasting period [10].

The patients undergoing elective surgery minor blood loss had fasted from midnight and were premedicated with 1 g of oral paracetamol. Surgeries included correction of pro- or retrognathia and breast reconstruction after ablatio. Chronic medications were tamoxifen (*N* = 3) and goserelin (*N* = 1) to treat breast cancer, and sumatriptan (*N* = 1) for migraine. General anesthesia without additional epidural analgesia was used as described in the original report [11]. A slow drip (0.5 mL/kg/h) of glucose 2.5% with electrolytes compensated for evaporation during the procedure. A low-dose infusion of norepinephrine (mean rate 2.6 µg/min) was infused intermittently.

The patients undergoing elective lower abdominal surgery for muscle invasive bladder cancer with major bleeding had fasted since midnight but received 400 mL oral standardised carbo-loading of a clear liquid solution in the morning before the surgery. Pre-exiting diseases included hypertension (*N* = 6), diabetes (*N* = 2), and chronic obstructive pulmonary disease (*N* = 2). Three patients had elevated plasma C-reactive protein concentration before surgery (10, 39 and 69 mg/L) but the remaining 7 patients had normal levels (< 5 mg/L). No premedication was given. Patients had combined low thoracic epidural and general anesthesia. Ringer´s lactate in the volume 1:1 with the surgical blood loss was given throughout the operations. An infusion of norepinephrine, starting at a rate of 0.03 µg kg^−1^ min^−1^, was given to maintain the mean arterial pressure (MAP) > 65 mmHg.

The postoperative patients were a population separated from the other two surgical groups. They were studied in the early morning, beginning 6.30 AM, after having undergone open abdominal surgery for treatment of cancer during the preceding day. The duration of the operations averaged 5.9 h and the perioperative blood loss was 700 mL. After midnight, no patient received colloid fluid in any form (i.e., no blood, plasma, albumin, or synthetic colloid). Hemodynamic stability was confirmed before starting the albumin infusion. All patients had a well-functioning epidural block and received a constant-rate infusion of ropivacaine 2 mg/mL plus sufentanil 0.5 µg/mL during the postoperative period [9]. Norepinephrine was given during the surgery, but not during the infusion experiment. No other fluid than 20% albumin was given.

Demographic and biochemical data are summarized in Table 1.

**Table 1:** Basic data on the studied subjects and data from the experiments

Variable	Volunteers	Post-burn	Surgery with minor hemorrhage	Surgery with major hemorrhage	Postoperative
*N*	15	15	15	10	15
Age (years)	31 (12)	45 (15)	46 (15)	66 (9)^a^	65 (13)^a^
Body weight (kg)	73 (14)	95 (17)^a^	72 (16)	79 (8)	73 (13)
Females/males	6/9	3/12	10/5	2/8	2/13
Infused fluid volume (mL)	229 (39)	281 (56)	216 (48)	238 (24)	218 (42)
*P*-albumin (g/L), baseline	41 (3)	24 (5)^a^	37 (2)	34 (4)	25 (5)^a^
MAP (mmHg), baseline	93 (6)	87 (1)	65 (6)	78 (6)	75 (8)
C-reactive protein (mg/L)	2 (3)	86 (20–294)	< 5^b^	62 (54–167)^c^	61 (21–216)
‘Inflammation’	No	Yes	No	Possible	Yes
Blood loss (mL)	140	140	340 (190–1340)	940 (640–1840)	None
*P*-creatinine (µmol/L)	78 (20)	78 (13)	71 (10)	105 (8)	67 (11)
Maximum *P*-dilution (%)	14.2 (4.8)	16.3 (6.0)	13.6 (5.6)	8.3 (5.6)^d^	13.3 (4.9)
Reference number	9, 10	10	11	12	9

Data are the mean (SD) but a few variables with skewed distribution are reported as the median (range)

^a^Significantly different from the other groups (Scheffé test *P* < 0.05); ^b^at the end of surgery, ^c^in the first postoperative morning; ^d^Ringer´s lactate was also infused

### Data collection

Blood (10 mL) was withdrawn at 15 exactly timed occasions over a period of 5 h after starting the infusion (0, 10, 20, 30, 40, 50, 60, 75, 90, 120, 150, 180, 210, 240 and 300 min). An exception was the "surgery with major hemorrhage" group where blood was not sampled at 210 min. Whole blood was analysed for hemoglobin (Hb) concentration and hematocrit (Hct). Plasma was used to measure the albumin concentration. The respective hospital´s certified laboratory was used for all clinical chemistry analyses.

The surgical blood loss was calculated based on the content of the suction bag and weighted sterile compresses. The reported blood loss is the sum of the surgical blood loss and the sampled blood (140 mL per experiment).

### Kinetic model

The kinetics of the infused albumin mass was analysed using a one-compartment model in which the elimination rate is proportional to the plasma albumin concentration at any time *t* (*C*_t_):

$$C_{{\text{t}}} = C_{{\text{o}}} e^{ - k\;t}$$

where *C*_o_ is the plasma concentration extrapolated to zero time and *k* the elimination rate constant.

The change in *C* at time *t* in response to an infusion given at the rate *R*_o_ is written as:

$${\text{d}}C/{\text{d}}t = R_{{\text{o}}} {-}kC_{{\text{t}}}$$

The present study focuses on an excess amount of albumin provided between Time 0 and Time t on top of the pre-existing intravascular albumin. However, the there is a mis-match between the change in the plasma albumin concentration (∆*P*-albumin_t−0_) and the intravascular albumin mass because 20% albumin attracts extravascular fluid by means of its high oncotic pressure [9–11]. This mismatch is accounted for by multiplying ∆*P*-albumin_t−0_ by the plasma dilution (*P*-dilution) at time *t*:

$${\text{d}}C/{\text{d}}t = R_{{\text{o}}} {-}k\left[ {\left( {\Delta P{\text{ - albumin}}_{{{\text{t}} - 0}} } \right){*}\left( {1 + P{\text{ - dilution}}_{{\text{t}}} } \right)} \right]$$

The plasma dilution at time *t* (*P*-dilution_t_) was calculated based on the decrease in the blood Hb concentration, which was then corrected for the baseline Hct to convert the data from dilution of whole blood to dilution of plasma; hence, ([Hb_0_/Hb_t_] − 1)/(1 − Hct_0_). This correction derives the ‘fictitious’ plasma concentration that corresponds to the intravascular albumin mass.

**Fig. 1:**
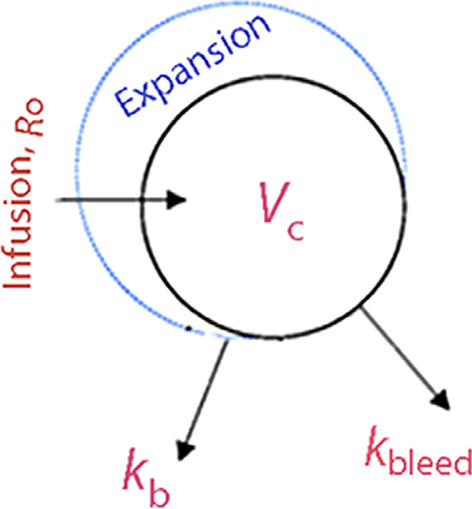
The kinetic model used for the analysis of albumin infused as a 20% solution in 70 subjects. Here, the elimination rate constant *k* is split into two functions, *k*_b_ and *k*_bleed_ where the former represents capillary leakage

A second elimination compartment was added later to separate the influence of hemorrhage-induced albumin loss from the total elimination (Fig. 1). Hence, the elimination rate constant *k* was split into two components, of which *k*_b_ represents the capillary leakage of albumin and *k*_bleed_ the known albumin losses by blood sampling and hemorrhage. The losses by hemorrhage were calculated for each subject as: (lost blood × (1 − mean hematocrit) × mean *P*-albumin) and, just as for the total elimination, the rate of the loss was modelled to increase in proportion to the dilution-corrected *P*-albumin.

In case the blood volume has changed due to a larger blood loss, a correction of the plasma dilution using mass balance is recommended to maintain the same volume of distribution for the infused albumin mass throughout the experiment. For this purpose, the blood volume at baseline (BV_0_) is estimated by one of the many available anthropometric equations [13, 14]. The total mass of Hb in the circulation is corrected for estimated Hb losses. A second blood Hb allows the calculation of BV at Time t (BV_t_) by the following series of mass balance calculations: [15]

$$\begin{aligned} & {\text{Hb}}_{{{\text{mass}}(0)}} = {\text{BV}}_{0} {\text{Hb}}_{0} \\ & {\text{BV}}_{{\text{t}}} = ({\text{Hb}}_{{{\text{mass}}(0)}} {-}{\text{Hb}}_{{{\text{loss}}({\text{t}} - 0)}} )/{\text{Hb}}_{{\text{t}}} \\ & {\text{PV}}_{0} = {\text{BV}}_{0} \left( {1{-}{\text{Hct}}_{0} } \right);\quad {\text{PV}}_{{\text{t}}} = {\text{BV}}_{{\text{t}}} (1{-}{\text{Hct}}_{{\text{t}}} ); \\ & {\text{Plasma dilution}} = {\text{PV}}_{{\text{t}}} /{\text{PV}}_{0} \\ \end{aligned}$$

Note that the baseline PV is placed in the denominator of the mass balance equation shown above, while the diluted Hb appears in the denominator of the hemodilution equation based on a decrease in Hb.

The bled volume is multiplied by the mean of Hb_0_ and Hb_t_ to obtain Hb_loss(t − 0)_.

### Kinetic analysis

The mono-exponential function was fitted to all 70 experiments simultaneously using the First Order Conditional Estimation Extended Least-Squares search routine in the Phoenix software for nonlinear mixed effects, version 8.3.4 (Pharsight, St. Louis, MO) and the additive model for the within-subject variability [16]. The curve-fitting estimated the fixed rate parameters *k*_b_ and *k*_bleed_ that determined the distribution of albumin, and also *V*_c_ which is a proportional factor between the administered albumin mass and the resulting increase of plasma albumin.

The initial curve-fitting was followed by covariate analysis, which implied that a kinetic parameter could modified due to the characteristics of one study group or an individual. Factors that were evaluated for covariance on a yes/no basis included moderately severe inflammation, ongoing surgery, and the postoperative setting. If a covariance is statistically significant the − 2 log-likelihood for the kinetic model is decreased by > 6.6 points. For example, if *k*_b_ in one group differs from the others, the modification has the form:

$$\begin{aligned}{\text{k}_{b}}({\text{specific}}\;{\text{group}})& = {\text{k}_{b}}({\text{all}}\;{\text{groups}})\\ &\quad*(2.718 \wedge {\text{covariate}}\;{\text{value}}) \end{aligned}$$

as 2.718 is the natural logarithm (e).

Age, body weight, and baseline plasma albumin were evaluated as possible modifiers (covariates) for each individual subject. As these data were continuous, a so-called “power model” was used that relates individual values to the population mean [16]. All covariance analyses were performed by using standard procedures in the Phoenix program.

A post hoc power analysis, based on the best estimate and standard error for all patients in the final kinetic model, shows that a difference in *k*_b_ of 20% would be able to distinguish 2 groups of 15 patients with a power of 90% based on *P* < 0.05.

The results are reported as mean ± standard deviation (SD) and kinetic parameters as the best (optimal) estimate and 95% confidence interval (CI). *P* < 0.05 was considered statistically significant.

## Results

### Study settings

The post-burn patients were studied 7 days (median, range 4–14 days) after their burn injury. The burn injury covered 15% (range 7–48%) of the total body surface [10].

The first group of surgical patients received 20% albumin over 30 min but no other fluid during the operation, and major hemorrhage was rare (median albumin loss 13 g, range 5–39) [11]. The second group of surgical patients suffered a larger hemorrhage that contained 30 g (mean, SD 11 g) of albumin; they were also given Ringer’s lactate continuously in addition to the standard infusion of 20% albumin over 30 min [12]. The postoperative patients were studied on the first morning after the major surgery [9].

Selected data on the study groups are given in Table 1. The patients who underwent surgery with major hemorrhage and the postoperative patients were significantly older than the others. The post-burn patients had the highest body weight, but they had received liberal acute volume loading just after their burn injury. The post-burn and the postoperative patients had lower plasma albumin than the other groups. All these selected between-group comparisons were made with one-way ANOVA (*P* < 0.001) followed by the Scheffé test (*P* < 0.05).

### Kinetic analyses

All 70 experiments were analysed on a single occasion. Seven samples points were incomplete, leaving 1033 paired measurements of plasma albumin and blood Hb concentrations to be analysed. The following four approaches were used:

1. The change in plasma albumin from baseline was analysed.
2. A covariate box plot suggested that *k* differed greatly between ‘Surgery with major hemorrhage’ and the other groups. Therefore, *k* in this group was adjusted according to a covariate effect, which means that the deviating *k* of these patients no longer affected the overall estimate of *k*.
3. The plasma albumin was corrected for the plasma volume expansion (as given by the Hb-derived plasma dilution) induced by the 20% albumin. In addition, *k* was split into two elimination functions that were governed by two rate constants, *k*_b_ and *k*_bleed_, which separated the unknown losses of intravascular albumin by transcapillary leakage from the known losses of albumin by hemorrhage and blood sampling (Fig. 1). Again, *k*_b_ in the group ‘Surgery with major hemorrhage’ served as a covariate.
4. Correction of plasma dilution for bleeding-induced changes of the distribution volume for albumin as indicated by the presented mass balance equations. The rationale behind this procedure is that the calculation of albumin losses based on the plasma albumin concentration requires a constant distribution volume.

### Final model

We regard the kinetic analysis No. 4 to be the most correct one. The reason is that No. 4 adjusts for all confounders that makes the setting for the analysis to deviate from the desired one, which is that the administered albumin should not change its own volume of distribution and that no losses of tracer should occur other than the one we intend to quantify.

**Fig. 2:**
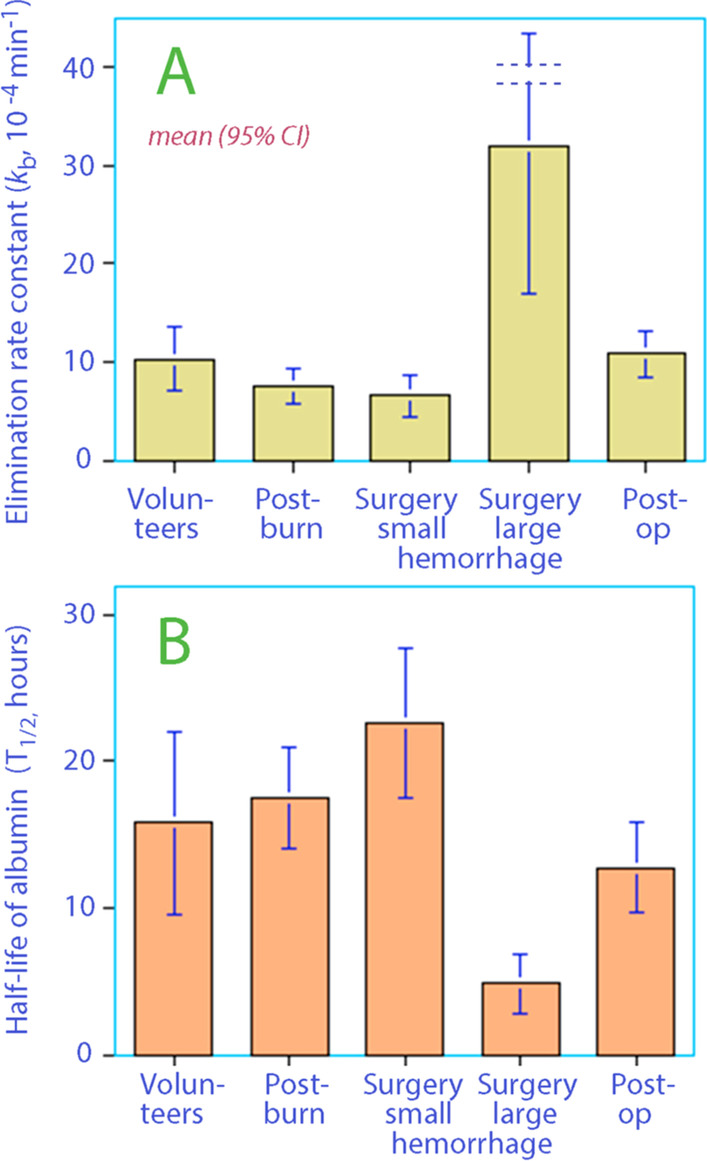
**A** Distribution of the elimination rate constant *k*_b_ between the five study groups. This constant represented the capillary leakage of infused excess albumin in the proposed kinetic model. **B** The corresponding half-lives of infused excess albumin

The box plot used for screening still suggested that *k*_b_ for the study groups was different in the ‘surgery with major hemorrhage’ group (Fig. 2). Therefore, *k*_b_ in this group was again treated as a covariate, whereby we accepted that *k*_b_ in those patients differed from that of the others.

**Fig. 3:**
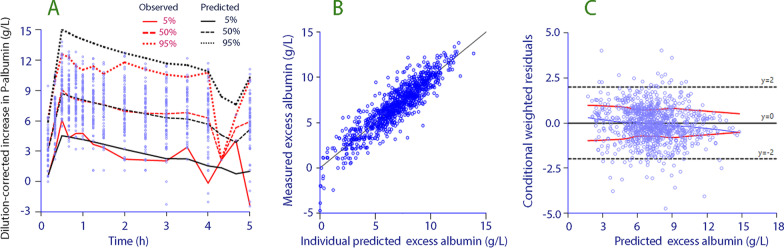
**A** Predictive check based on 1000 runs. **B** Goodness of fit of the kinetic model; predicted *versus* measured dilution-corrected plasma albumin concentrations, and **C** conditional weighted residuals *versus* predicted values. The thin blue line shows the logically weighted scatterplot smoothing line for the residuals; the regular red lines indicate the logically weighted scatterplot smoothing for the absolute residuals, and the irregular black lines show ± 2 SD for the residuals

Figure 3 Illustrates the performance and goodness of fit of the final model

Further covariate analyses confirmed that the ‘inflammation’ (either 2/5 or 3/5 of the groups, see Table 1) and ‘postoperative setting’ could not serve as statistically significant covariates of the rate constant describing capillary leakage of albumin (*k*_b_). The individual data on age, body weight, and the baseline plasma albumin had no statistically significant effect on *k*_b_. This means that the likelihood for the kinetic model did not improve by including them, which was done in sequence.

## Discussion

### Key results

Several clinical settings commonly believed to be associated with endothelial damage did not have a short T_1/2_ for exogenous albumin. Albumin given in moderately severe inflammatory states, during surgery without major hemorrhage and during postoperative recovery after major surgery disappeared at rates that were not statistically different from the T_1/2_ measured in healthy volunteers. By contrast, surgery with major hemorrhage had a markedly shorter intravascular persistence.

The results also illustrate that the T_1/2_ may differ greatly depending on whether the calculations consider plasma volume changes and blood loss.

### Interpretation

The intravascular T_1/2_ for excess albumin is relevant to the clinical efficacy of 20% albumin solution, as every gram of albumin binds 10–11 mL of water in the plasma [6]. The fluid content of the 20% albumin preparation is only 5 mL per gram albumin, which means that an infusion recruits fluid from extravascular sources to the plasma. The maximum plasma volume expansion is approximately twice the infused volume [9–12, 17, 18].

The T_1/2_ of 16.2 h in our final model corresponds to a loss of 3.8% of the plasma pool of albumin per h. This is a long T_1/2_ and might reflect that exogenous albumin limits capillary leakage of albumin, but this hypothesis, which has been suggested by laboratory experiments, has not been confirmed in septic patients [19].

The surgical group with major hemorrhage lost approximately 15% of the intravascular albumin pool per h (T_1/2_ 4.9 h). This accelerated rate can be real, but it can also be due to underestimation of the albumin losses during the surgery. A margin of error could be present even if blood loss was assessed to the best of the clinical disposable options. All operations were open cystectomies, which may be associated with widespread exudation from open body surfaces. In addition, these patients also underwent extended lymph node dissection which, like surgery for ovarian cancer [20], might be associated with increased albumin losses.

### Literature

The normal capillary leakage rate for albumin is 5% per h (T_1/2_ 10 h) [1, 17]. Data from surgery and intensive care show greater variability. Fleck et al*.* found doubled capillary leakage rates 7 h after cardiac surgery and quadrupled rates in patients with septic shock [2]. T_1/2_ was 7.7 h in another study of sepsis [19], and as short as 3.6 h in peritoneal dialysis patients [21]. Norberg et al*.* found a normal T_1/2_ of 10 h immediately before and two days after pancreatic resection [22] but a T_1/2_ of 6.3 h during the actual surgery [23].

Studies that agree best with our current result include a mass balance study showing a T_1/2_ corresponding to 17 h during major surgery [24] and a T_1/2_ of 16 h as derived by radioisotopes corrected for plasma dilution after coronary bypass surgery [18].

### Methodology

Assessments of albumin kinetics are hampered by losses of the biomarker with protein exudation and blood loss, which have only occasionally been considered in the literature.

Our aim in analysing albumin kinetics was to handle three confounding effects: the dilution caused by volume loading, the change of the volume of distribution for intravascular albumin associated with blood withdrawal or hemorrhage and, finally, the loss of albumin due to hemorrhage and protein exudation.

The first two mathematical corrections derived the fictious plasma albumin concentration that would prevail if the volume of distribution for the albumin mass (i.e., the plasma volume) had been unchanged during the study period. A fixed volume of distribution is a prerequisite for allowing the plasma concentration to indicate an eliminated amount of albumin.

The first challenge was the easiest to deal with because the dilution of the plasma can be calculated using Hb as an endogenous tracer. The second transformation involved a more cumbersome changeover to mass balance calculations. The first correction moves the concentration time curve upwards, while the second one depresses the curve. The correction for blood loss separates the elimination into two components and lifts the concentration–time curve upwards to indicate a longer T_1/2_. Hence, inability to account for blood loss leads us to believe that the transcapillary leakage of albumin occurs faster than it does.

The correction of plasma albumin for plasma dilution is rarely crucial for the kinetics of exogenous albumin, except for large hemorrhage volumes. The reason is that the increase in plasma albumin varies from about 1 to 10 g/L (i.e., tenfold), whereas the plasma dilution changes only by 10–20%. The last step, the separation of known albumin losses into a second elimination compartment, had a much greater influence on the T_1/2_.

How the modifications of the kinetic analysis gradually change T_1/2_ can be reviewed in Table 2. Our four modes of calculating the T_1/2_ illustrate that considerably different results are obtained depending on how these methodological problems are handled. The same issues will be confounders when using isotopes to assess capillary leakage of albumin as well (Table 3).

**Table 2:** Population kinetic parameters for albumin administered as a 20% solution in 70 subjects

Kinetic parameter	Best estimate	95% CI	CV%	T_1/2_ (h)
**1. Plasma albumin increase only**				
tv*V*_c_ (L)	5.88	5.51–6.26	3.2	
tv*k* (10^−4^ min^−1^)	13.8	9.9–17.6	14.3	8.4 (6.6–11.6)
**2. Albumin increase corrected for outliers**				
tv*V*_c_ (L)	5.87	5.50–6.23	3.3	
tv*k* (10^−4^ min^−1^)	10.7	8.2–13.2	12.0	10.8 (8.7–14.1)
Surgery with major hemorrhage, covariate (0/1)^a^	2.30	1.88–2.72	9.2	
**3. Albumin × plasma dilution + compartment for albumin losses**				
tv*V*_c_ (L)	5.11	4.81–5.42	3.1	
tv*k*_b_ (10^−4^ min^−1^)	5.80	5.04–6.57	6.7	19.9 (17.6–22.9)
tv*k*_bleed_ (10^−4^ min^−1^)	5.58	4.82–6.35	7.0	
Surgery with major hemorrhage, covariate (0/1)^a^	1.37	1.12–1.62	9.3	
**4. Plasma dilution corrected for bleeding-induced changes in distribution volume**				
tv*V*_c_ (L)	5.17	4.86–5.48	3.0	
tv*k*_b_ (10^−4^ min^−1^)	7.12	6.04–8.21	7.8	16.2 (14.0–19.1)
tv*k*_bleed_ (10^−4^ min^−1^)	5.81	4.82–6.79	8.6	
Surgery with major hemorrhage, covariate (0/1)^a^	1.38	1.06–1.70	11.7	

Four different approaches are shown for the calculation of the intravascular half-life (T_1/2_), but only the last two consider the shortening that occurs due to blood sampling and surgery-induced blood loss

tv = typical value for the group. CI = confidence interval. CV% = coefficient of variation (inter-individual)

^a^Covariate means that the *k*_b_ in that group becomes tv*k*_b_ = tv*k*_b_ * (2.178 ^ covariate value) and others only tv*k*_b_

**Table 3:** Pharmacokinetic parameters in the studied groups based on individual estimates derived *post hoc*

Variable	Volunteers	Post-burn	Surgery, minor hemorrhage	Surgery, major hemorrhage	Postoperative
*V*_c_ (L)^a^	6.2 (5.2–7.1)^b^	5.9 (5.4–6.4)	5.1 (4.5–5.7)	4.3 (4.2–5.0)	4.6 (4.2–5.0)
tv*k*_b_ (10^−4^ min^−1^)	10.2 (7.0–13.5)	7.5 (5.8–9.2)	6.5 (4.3–8.7)	32.0 (16.8–47.4)^c^	10.8 (8.3–13.2)
tv*k*_bleed_ (10^−4^ min^−1^)	3.4 (2.7–4.1)	2.2 (2.0–2.5)	14.0 (8.7–19.4)	88.8 (40.8–136.8)^c^	3.4 (2.9–3.8)
T_1/2_ (h)	15.8 (9.6–22.0)	17.5 (14.1–21.0)	22.6 (17.5–27.7)	4.9 (2.8–6.9)^c^	12.7 (9.7–15.8)

Data are the mean (95% CI) and derived post hoc based on the population kinetic analysis

^a^Calibration factor between the infused albumin mass and the increase of the plasma albumin concentration

^b^Differed from the two last groups *P* < 0.05 (Scheffé test) after one-way ANOVA *P* < 0001

^c^Differed from all other groups by *P* < 0.05 (Scheffé test) after one-way ANOVA *P* < 0001

### Implications

Our results suggest that more attention should be paid to the circumstances under which capillary leakage is measured under non-steady state conditions, such as during surgery and in severe disease. They also imply that a modestly severe trauma is not enough the increase the capillary leakage of exogenous albumin. The clinician does not have to reckon with short duration or poor intravascular expansion from infused 20% albumin if used within the limits presented in this report.

### Limitations

The reported T_1/2_ is not identical to the capillary leakage rate because albumin is recirculated to the plasma via lymphatic flow. The intravascular albumin pool is normally at a steady state with the inflow; therefore, the T_1/2_ be used as an estimate of the ‘true’ capillary leakage rate of exogenous albumin only if this steady state is maintained. Possible imbalances between the rate of albumin synthesis and catabolism also have to be overlooked.

The present report is based on pooled data from four published studies where the same protocol has been used [9–12]. The two studies with established inflammation were all associated with plasma C-reactive protein concentrations of 60–80 mg/L, which is commonly encountered after major surgery or in patients with advanced cancer stages. This concentration is exceeded in severe inflammatory states [22], which may increase the capillary leakage of albumin.

The elevation of C-reactive protein during cancer surgery with major hemorrhage is more uncertain, as the concentration reported in Table 1 was measured in the first postoperative morning. Only three of the cancer patients had elevated C-reactive protein concentration before the surgery. However, the patients undergoing cancer surgery probably had a gradually intensified inflammatory response during the operations.

An unsolved issue is how the “normal” T_1/2_ in the post-burn and postoperative patients agrees with the low plasma albumin measured at baseline in these groups. Further studies will be needed to separate the contributions of accelerated capillary leakage, retarded lymphatic recirculation, and plasma dilution to this depression of plasma albumin. If explained by dilution alone, the low plasma albumin in these groups would require a plasma volume expansion of 2 L.

## Conclusion

No accelerated disappearance rate was observed when albumin was given as a 20% solution in patients with inflammation due to post-burn injury, in patients undergoing surgery without major hemorrhage, or in patients studied one day after major surgery. In these settings, infused albumin remained in the plasma as long as was observed in volunteers.

## Supplementary Information

**Additional file 1:** The original data used for the kinetic analysis.

## Data Availability

All individual data are given in Additional file 1.

## Abbreviations

BV: Blood volume
Hb: Hemoglobin
Hct: Hematocrit
*k*_b_: Rate constant for capillary leakage of albumin
*k*_bleed_: Rate constant for albumin losses with blood sampling and surgical bleeding
PV: Plasma volume
T_1/2_: Half-life
*V*_c_: Volume of distribution, which is the scaling factor between the added intravascular amount of albumin and resulting change of the plasma concentration
